# Type Ib Abernethy Malformation Complicated by Hepatopulmonary Syndrome: A Case Report of a Rare Entity in a Pediatric Patient

**DOI:** 10.7759/cureus.111965

**Published:** 2026-07-02

**Authors:** Rohan S Pardeshi, Kajal Mitra, Mayank Rangari, Prashant Onkar, Suresh Phatak

**Affiliations:** 1 Department of Radiology, N. K. P. Salve Institute of Medical Sciences and Research Centre and Lata Mangeshkar Hospital, Nagpur, IND

**Keywords:** abernethy malformation, congenital extrahepatic portosystemic shunt, ct angiography, hepatopulmonary syndrome, intrapulmonary vascular dilatation, liver transplantation, pediatric cyanosis, pediatric radiology, portal vein agenesis, portosystemic shunt

## Abstract

Abernethy malformation is a rare congenital extrahepatic portosystemic shunt (CEPSh) characterized by the diversion of portal venous blood away from the liver into the systemic circulation, resulting in a wide spectrum of hepatic, pulmonary, and systemic complications. Hepatopulmonary syndrome is an important but uncommon association that results from intrapulmonary vascular dilatation secondary to portosystemic shunting. We describe the case of a two-year-old female who was evaluated for recurrent respiratory tract infections, cyanosis, and digital clubbing. Radiologic assessment demonstrated a Type Ib Abernethy malformation characterized by the formation of a common spleno-mesenteric trunk draining directly into the inferior vena cava, complete diversion of portal venous flow, and the absence of intrahepatic portal venous radicals. CT pulmonary angiography further revealed dilated distal pulmonary vasculature compatible with hepatopulmonary syndrome. Prompt radiologic recognition of this rare vascular anomaly is crucial, particularly when pulmonary manifestations are present, as timely diagnosis and referral for definitive management, including liver transplantation, can significantly improve clinical outcomes.

## Introduction

Abernethy malformation, also known as congenital extrahepatic portosystemic shunt (CEPSh), is a rare vascular anomaly characterized by the abnormal diversion of portal venous blood away from the liver into the systemic venous circulation. In this condition, blood from the gastrointestinal tract, spleen, and pancreas bypasses the hepatic sinusoids either partially or completely, thereby impairing normal hepatic metabolism and detoxification of portal blood. The anomaly arises due to abnormalities during the embryological development of the vitelline venous system, resulting in the persistence of abnormal portosystemic communications and incomplete formation of the portal venous network. Although uncommon, Abernethy malformations are clinically significant because of their association with multiple hepatic, cardiovascular, pulmonary, metabolic, and neurodevelopmental abnormalities [1].

The classification proposed by Morgan and Superina remains the most widely accepted system for categorizing CEPSh. According to this classification, CEPSh are divided into Type I and Type II malformations based on the degree of diversion of portal blood flow to the systemic circulation. Type I shunts represent complete diversion of portal venous blood away from the liver, with congenital absence of intrahepatic portal venous branches. In contrast, Type II shunts are characterized by partial diversion of portal flow, wherein a hypoplastic but patent intrahepatic portal venous system is present, and a portion of portal blood continues to perfuse the liver [1,2].

Type I malformations are further subdivided into Type Ia and Type Ib depending on the anatomical configuration of the splenic vein (SV) and superior mesenteric vein (SMV). In Type Ia shunts, SV and SMV drain separately into the systemic venous circulation without forming a confluence. In Type Ib malformations, SV and SMV first unite to form a common spleno-mesenteric trunk, which subsequently drains directly into a systemic vein, most commonly the inferior vena cava. This subtype is associated with complete absence of intrahepatic portal venous branches and total diversion of portal venous blood away from the liver [2,3].

The absence of normal portal venous perfusion in Type I Abernethy malformation has important physiological and clinical consequences. Portal blood contains hepatotrophic factors essential for normal hepatic growth, regeneration, and metabolic function. Lack of portal perfusion may therefore result in hepatic atrophy, nodular regenerative hyperplasia, focal nodular hyperplasia, hepatic adenomas, or even hepatocellular carcinoma. Furthermore, the systemic circulation receives substances that would normally undergo hepatic clearance, predisposing affected individuals to complications such as hepatic encephalopathy, hepatopulmonary syndrome, and pulmonary arterial hypertension. Associated congenital anomalies involving the cardiovascular, gastrointestinal, genitourinary, and skeletal systems are also frequently reported, particularly in patients with Type I shunts [3].

Advances in cross-sectional imaging, especially Doppler ultrasonography, CT, and MRI, have significantly improved the diagnosis and characterization of Abernethy malformations. Imaging plays a crucial role in identifying the anatomy of the shunt, assessing the presence or absence of intrahepatic portal venous branches, detecting associated hepatic lesions, and guiding therapeutic planning. Accurate differentiation between Type I and Type II shunts is essential because management strategies differ considerably, ranging from shunt closure in selected Type II lesions to liver transplantation in symptomatic patients with Type I malformations [1-3].

## Case presentation

A two-year-old female child was brought with complaints of high-grade fever of three days’ duration, associated with a history of recurrent respiratory tract infections since early childhood. The recurrent infections were predominantly characterized by cough, fever, and respiratory distress, necessitating repeated medical consultations. There was no history of jaundice, altered sensorium, gastrointestinal bleeding, abdominal distension, or failure to thrive. Additionally, there was no prior history suggestive of chronic liver disease or congenital heart disease.

On clinical examination, the child was conscious, active, and hemodynamically stable. General physical examination revealed the presence of digital clubbing and peripheral cyanosis. Oxygen saturation measured on room air was 97%. There was no evidence of icterus, pallor, lymphadenopathy, pedal edema, or signs of chronic liver disease. Abdominal examination did not reveal hepatomegaly, splenomegaly, ascites, or any palpable abdominal mass. Cardiovascular system examination did not demonstrate any significant murmur or evidence of cardiac failure. Respiratory system examination was unremarkable except for findings related to recurrent respiratory tract involvement.

Baseline laboratory investigations, including complete blood count, liver function tests, renal function tests, coagulation profile, and serum ammonia levels, were within normal limits. No biochemical evidence of hepatic dysfunction or hyperammonemia was identified at presentation. In view of cyanosis and recurrent respiratory symptoms, a detailed cardiopulmonary evaluation was undertaken. Two-dimensional echocardiography revealed no structural cardiac abnormality or intracardiac shunt. Subsequently, contrast-enhanced CT pulmonary angiography was performed, which demonstrated marked dilatation of segmental and subsegmental pulmonary vessels with diffuse peripheral vascular prominence, findings consistent with hepatopulmonary syndrome secondary to an underlying portosystemic shunt (Figure 1).

**Figure 1:**
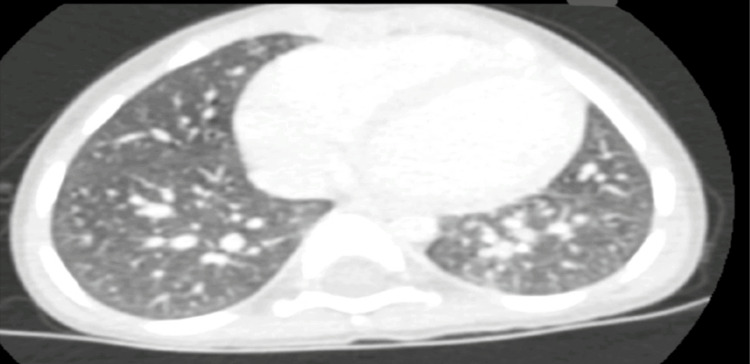
CT pulmonary angiography (axial lung window) The image shows dilated segmental and subsegmental pulmonary veins, suggestive of hepatopulmonary syndrome CT: computed tomography

Further evaluation with contrast-enhanced CT angiography of the abdomen demonstrated a congenital extrahepatic portosystemic shunt consistent with Type Ib Abernethy malformation (Figures 2, 3). The splenic vein and superior mesenteric vein were seen forming a common spleno-mesenteric trunk, which drained directly into the inferior vena cava. There was complete diversion of portal venous blood into the systemic venous circulation with non-visualization of intrahepatic portal venous radicals, indicating congenital absence of the intrahepatic portal venous system.

**Figure 2:**
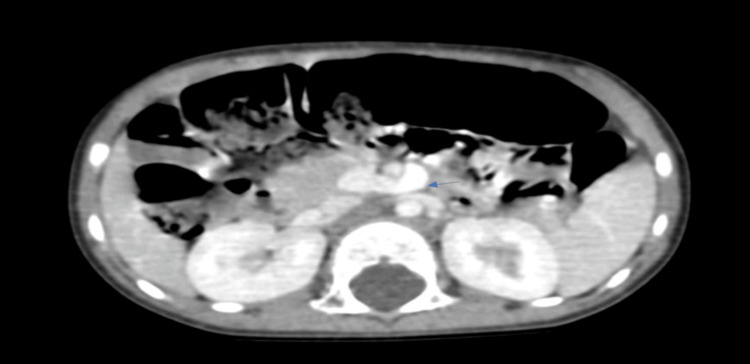
Contrast-enhanced CT abdomen (axial) The image shows the splenic vein and superior mesenteric vein forming a common channel posterior to the superior mesenteric artery (blue arrow) CT: computed tomography

**Figure 3:**
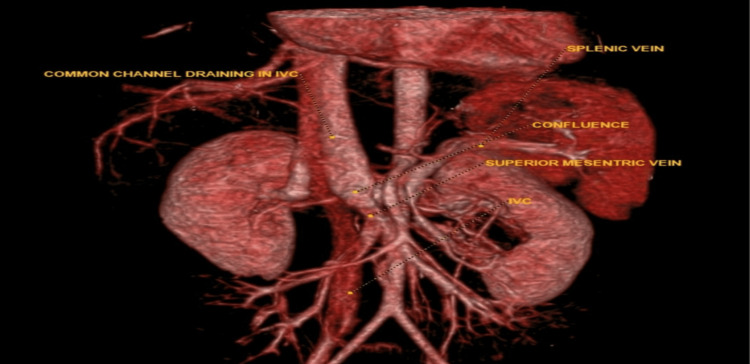
Three-dimensional volume-rendered CT angiography The image demonstrates a common portal venous channel draining directly into the inferior vena cava, consistent with Type Ib Abernethy malformation CT: computed tomography

Ultrasonography of the abdomen with color Doppler correlation confirmed the CT findings (Figures 4, 5). An anomalous venous channel was identified draining the spleno-mesenteric confluence directly into the inferior vena cava. The portal vein was not visualized at the porta hepatis (Figure 6). These imaging findings established the diagnosis of Type Ib Abernethy malformation associated with hepatopulmonary syndrome.

**Figure 4:**
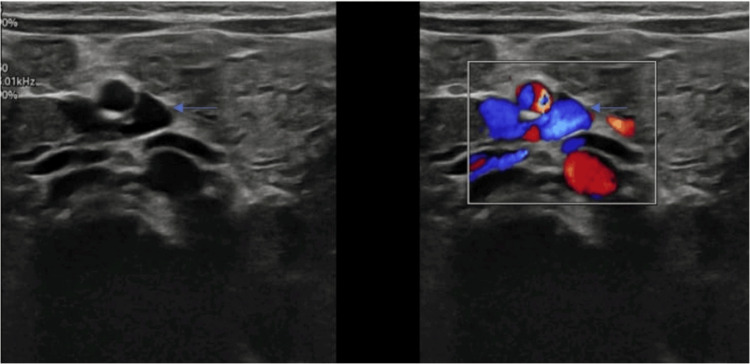
Gray-scale and color Doppler ultrasonography The images demonstrate the splenic vein and superior mesenteric vein forming a common channel posterior to the superior mesenteric artery (blue arrow)

**Figure 5:**
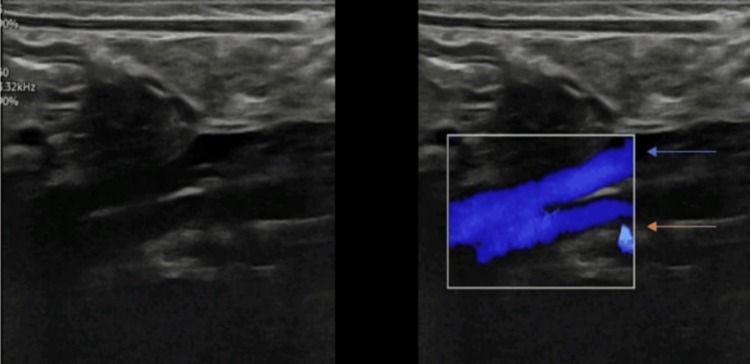
Color Doppler ultrasonography The images show a common portal venous channel draining directly into the inferior vena cava (blue arrow: inferior vena cava; orange arrow: common portal channel)

**Figure 6:**
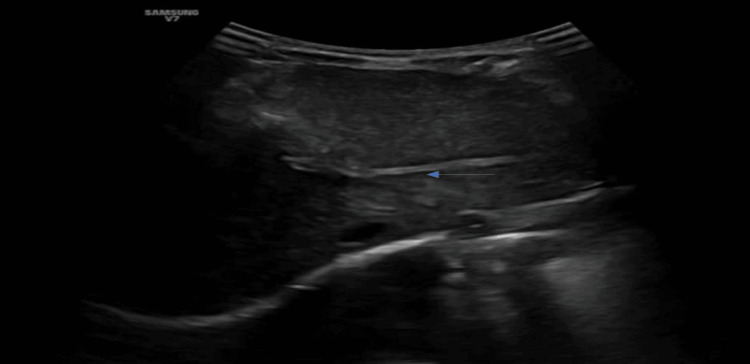
Gray-scale ultrasonography The image shows an atretic/hypoplastic intrahepatic portal venous system (blue arrow)

## Discussion

Abernethy malformation is an extremely rare congenital vascular anomaly resulting from abnormal embryologic development of the vitelline venous system between the fourth and 10th weeks of gestation [1]. Failure of normal remodeling of these veins leads to persistence of anomalous porto-systemic channels, allowing splanchnic venous blood to bypass the liver. Type I shunts account for a minority of reported cases and are associated with complete absence of intrahepatic portal venous supply [2,3]. Clinical manifestations depend on shunt type, size, and duration of portal blood diversion. Type I shunts typically present early in life with metabolic disturbances, hepatic dysfunction, pulmonary vascular complications, or the development of hepatic nodules [4]. Hepatopulmonary syndrome is a recognized complication and results from intrapulmonary vascular dilatation caused by circulating vasodilators, particularly nitric oxide, that bypass hepatic metabolism [5,6].

Imaging plays a pivotal role in diagnosis and classification of congenital extrahepatic portosystemic shunts. Ultrasonography with color Doppler serves as an initial screening modality, while contrast-enhanced CT or MR angiography provides definitive delineation of shunt anatomy and assessment of intrahepatic portal venous branches [7]. Differentiation from acquired causes such as chronic portal vein thrombosis is essential, as congenital shunts are not associated with cavernous transformation or features of portal hypertension [8-10]. Accurate radiologic differentiation between Type I and Type II shunts is crucial, as it directly influences therapeutic decisions. While Type II shunts may be amenable to endovascular or surgical closure, Type I shunts lack intrahepatic portal venous branches and therefore do not tolerate shunt occlusion. These patients typically require liver transplantation, particularly when complicated by hepatopulmonary syndrome [11,12]. Early diagnosis is essential, as prolonged hypoxemia may lead to irreversible pulmonary vascular remodeling.

## Conclusions

A two-year-old female child presented with high-grade fever for three days and a history of recurrent respiratory tract infections since early childhood. Clinical examination revealed digital clubbing and peripheral cyanosis, with oxygen saturation of 97% on room air. There was no icterus, hepatosplenomegaly, ascites, or other signs of chronic liver disease. Cardiovascular examination was unremarkable. Laboratory investigations including complete blood count, liver function tests, renal function tests, coagulation profile, and serum ammonia levels were within normal limits, with no evidence of hepatic dysfunction or hyperammonemia. In view of recurrent respiratory symptoms and cyanosis, further evaluation was performed. Two-dimensional echocardiography showed no structural cardiac abnormality or intracardiac shunt. Contrast-enhanced CT pulmonary angiography demonstrated dilated segmental and subsegmental pulmonary vessels, suggestive of hepatopulmonary syndrome. Contrast-enhanced CT angiography of the abdomen revealed a Type Ib Abernethy malformation in which the splenic vein and superior mesenteric vein formed a common spleno-mesenteric trunk draining directly into the inferior vena cava, with complete absence of intrahepatic portal venous radicals. Ultrasonography with color Doppler confirmed non-visualization of the portal vein at the porta hepatis and demonstrated an anomalous venous channel draining the spleno-mesenteric confluence into the inferior vena cava.

This case report emphasizes the importance of considering congenital portosystemic shunts in children presenting with unexplained cyanosis and recurrent respiratory symptoms in the absence of cardiac or primary pulmonary pathology. Early recognition through appropriate imaging is crucial, as these anomalies may be associated with significant complications, including hepatopulmonary syndrome, and require timely multidisciplinary evaluation to guide further management, including consideration of definitive interventions such as liver transplantation in selected cases.

## References

[1] Abernethy J (1793). Account of two instances of uncommon formation, in the viscera of the human body. Phil Trans R Soc.

[2] Morgan G, Superina R (1994). Congenital absence of the portal vein: two cases and a proposed classification system for portasystemic vascular anomalies. J Pediatr Surg.

[3] Howard ER, Davenport M (1997). Congenital extrahepatic portocaval shunts—the Abernethy malformation. J Pediatr Surg.

[4] Alonso-Gamarra E, Parrón M, Pérez A, Prieto C, Hierro L, López-Santamaría M (2011). Clinical and radiologic manifestations of congenital extrahepatic portosystemic shunts: a comprehensive review. Radiographics.

[5] Grace JA, Angus PW (2013). Hepatopulmonary syndrome: update on recent advances in pathophysiology, investigation, and treatment. J Gastroenterol Hepatol.

[6] Ghent CN, Levstik MA, Marotta PJ (2008). The hepatopulmonary syndrome. N Engl J Med.

[7] Gupta P, Sinha A, Sodhi KS, Lal A, Debi U, Thapa BR, Khandelwal N (2015). Congenital extrahepatic portosystemic shunts: spectrum of findings on ultrasound, computed tomography, and magnetic resonance imaging. Radiol Res Pract.

[8] Bernard O, Franchi-Abella S, Branchereau S, Pariente D, Gauthier F, Jacquemin E (2012). Congenital portosystemic shunts in children: recognition, evaluation, and management. Semin Liver Dis.

[9] Stringer MD (2008). The clinical anatomy of congenital portosystemic venous shunts. Clin Anat.

[10] Sarin SK, Philips CA, Kamath PS, Choudhury A, Maruyama H, Nery FG, Valla DC (2016). Toward a comprehensive new classification of portal vein thrombosis in patients with cirrhosis. Gastroenterology.

[11] Franchi-Abella S, Gonzales E, Ackermann O, Branchereau S, Pariente D, Guérin F (2018). Congenital portosystemic shunts: diagnosis and treatment. Abdom Radiol (NY).

[12] Warner S, McKiernan PJ, Hartley J (2018). Hepatopulmonary syndrome in children: a 20‐year review of presenting symptoms, clinical progression, and transplant outcome. Liver Transpl.

